# Magnetoelectric phase transition driven by interfacial-engineered Dzyaloshinskii-Moriya interaction

**DOI:** 10.1038/s41467-021-25759-1

**Published:** 2021-09-15

**Authors:** Xin Liu, Wenjie Song, Mei Wu, Yuben Yang, Ying Yang, Peipei Lu, Yinhua Tian, Yuanwei Sun, Jingdi Lu, Jing Wang, Dayu Yan, Youguo Shi, Nian Xiang Sun, Young Sun, Peng Gao, Ka Shen, Guozhi Chai, Supeng Kou, Ce-Wen Nan, Jinxing Zhang

**Affiliations:** 1 Department of Physics, Beijing Normal University, 100875 Beijing, PR China; 2 Key Laboratory for Magnetism and Magnetic Materials of the Ministry of Education, Lanzhou University, 730000 Lanzhou, PR China; 3 International Center for Quantum Materials, Peking University, 100871 Beijing, PR China; 4 Electron Microscopy Laboratory, School of Physics, Peking University, 100871 Beijing, PR China; 5 Beijing National Laboratory for Condensed Matter Physics, Institute of Physics, Chinese Academy of Sciences, 100190 Beijing, PR China; 6 School of Physical Science, University of Chinese Academy of Sciences, 100190 Beijing, PR China; 7 School of Materials Science and Engineering, Tsinghua University, 100084 Beijing, PR China; 8 Advanced Research Institute of Multidisciplinary Science, Beijing Institute of Technology, 100081 Beijing, PR China; 9 Department of Electrical and Computer Engineering, Northeastern University, 02115 Boston, MA USA; 10 Collaborative Innovation Centre of Quantum Matter, 100871 Beijing, PR China

**Keywords:** Ferroelectrics and multiferroics, Surfaces, interfaces and thin films

## Abstract

Strongly correlated oxides with a broken symmetry could exhibit various phase transitions, such as superconductivity, magnetism and ferroelectricity. Construction of superlattices using these materials is effective to design crystal symmetries at atomic scale for emergent orderings and phases. Here, antiferromagnetic Ruddlesden-Popper Sr_2_IrO_4_ and perovskite paraelectric (ferroelectric) SrTiO_3_ (BaTiO_3_) are selected to epitaxially fabricate superlattices for symmetry engineering. An emergent magnetoelectric phase transition is achieved in Sr_2_IrO_4_/SrTiO_3_ superlattices with artificially designed ferroelectricity, where an observable interfacial Dzyaloshinskii-Moriya interaction driven by non-equivalent interface is considered as the microscopic origin. By further increasing the polarization namely interfacial Dzyaloshinskii-Moriya interaction via replacing SrTiO_3_ with BaTiO_3_, the transition temperature can be enhanced from 46 K to 203 K, accompanying a pronounced magnetoelectric coefficient of ~495 mV/cm·Oe. This interfacial engineering of Dzyaloshinskii-Moriya interaction provides a strategy to design quantum phases and orderings in correlated electron systems.

## Abstract (web summary)

A controllable magnetoelectric phase based on symmetry engineering is challenge in natural bulk crystals. Here, via engineering of interfacial Dzyaloshinskii-Moriya interaction, the authors design a magnetoelectric phase transition in oxide superlattices of correlated electron systems.

## Introduction

**Fig. 1:**
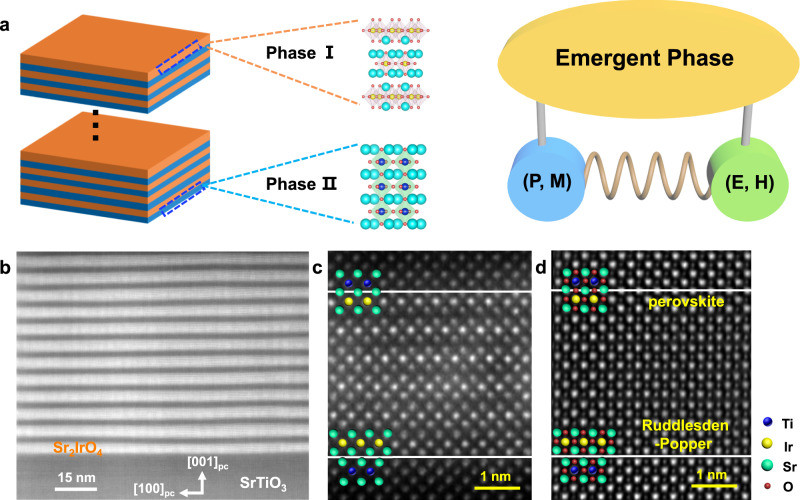
Emergent phase by the artificial design of oxide superlattices. **a** Creating emergent phases by an artificial assembly of two intrinsic functional phase I and II in oxide superlattices illustrated with simultaneous manipulation of spin (**M**), polar (**P**), even effective electric (**E**) or magnetic fields (**H**). **b** High angle annular dark-field (HAADF)-scanning transmission electron microscopy (STEM) images taken along the STO [010] zone axis at room temperature. **c**, **d** Atomically resolved HAADF and integrated differential phase contrast (iDPC) images, where the non-equivalent interfaces were constructed in the (Sr_2_IrO_4_)_3_/(SrTiO_3_)_6_ (I_3_/T_6_) superlattice, antiferromagnetic Sr_2_IrO_4_ (SIO) and dielectric SrTiO_3_ (STO) have been selected as functional Phase I and II.

Symmetry engineering is crucial to artificially design emergent phase transitions and accompanying controllable functionalities in condensed matters, especially in strongly correlated oxides^1–9^. Atomic construction of oxide interfaces and heterostructures is an effective method to break crystals’ intrinsic symmetries and create classical and quantum phase transitions^10–16^. Oxide superlattices provide a versatile playground to engineer the abundant interfacial structures to break space-inversion or time-reversal symmetry^17,18^. Therefore, modified or even emergent phases^19–25^ (exotic polar and (anti)ferromagnetic phases, etc.) may be created by an artificial assembly of intrinsic functional phase I and II as shown in Fig. 1a. In the recent decade, simultaneous and concurrent breaking of space-inversion and time-reversal symmetries seem to produce not only topological-protected states^26,27^ or nonlinear magneto-optical effects^28^, but also intrinsic magnetoelectric (ME) effects in the ferrotoroidal^29^ and axion insulator^30^, which are accompanied with the potential applications in future integrated information processing with low-energy consumption^4,28,31–35^. Therefore, it is highly desirable to design a controllable ME phase based on symmetry engineering and the interplay of quantum orderings in artificial superlattices, which is absent or rare in natural bulk crystals^28,31,35^.

Dzyaloshinskii–Moriya interaction (DMI) has been considered to be an effective energy to generate spiral spin textures via antisymmetric magnetic couplings in non-centrosymmetric (anti)ferromagnets with spin–orbit coupling, which bridges the time-reversal symmetry with space-inversion symmetry^36–38^. Emergent order parameters or phase transitions with ME effects may be driven by simultaneous manipulation of spin (**M**), polar (**P**), even effective electric (**E**), or magnetic fields (**H**) via engineering the DMI at atomic scale^39^. Artificial design of superlattices by alternately stacking Ruddlesden–Popper (A_*n* + 1_B_*n*_O_3*n* + 1_) and perovskite (ABO_3_) oxides is an effective way to manipulate space-inversion symmetry at atomic scale^18^ and thus gives a prerequisite for emergent DMI^36,37,40^. Among the Ruddlesden–Popper oxides, Mott insulator Sr_2_IrO_4_ (SIO) exhibits strong spin–orbit coupling and antiferromagnetism^41–43^. The perovskite SrTiO_3_ (STO) is a paraelectric oxide that could be readily manipulated to be ferroelectric with moderate thermodynamic variables^44,45^. Thus, the magnetic system with strong spin–orbit coupling and polar structure in the inversion-symmetry-broken superlattices by combining SIO and STO can be regarded as potential ingredients to the DMI^34,35^.

In this work, antiferromagnetic Ruddlesden–Popper SIO and perovskite paraelectric STO with a compatible growth condition is selected to epitaxially fabricate superlattices, where a non-equivalent interface is constructed to break the space-inversion symmetry. In the SIO/STO superlattices with artificially designed ferroelectricity, an emergent ME phase transition is achieved, which is driven by the interfacial DMI. By replacing paraelectric STO with ferroelectric BaTiO_3_ (BTO) to further engineer this interfacial DMI, the transition temperature can be enhanced from 46 to 203 K.

## Results

### Artificial design of oxide superlattices with non-equivalent interfaces

SIO and STO have been selected as functional Phase I and II in Fig. 1a. A series of SIO/STO superlattices have been grown on STO (001) single-crystal substrates (see Methods and Supplementary Fig. 1). A layer-by-layer growth at the atomic scale can be achieved for (SIO)_m_/(STO)_n_ (I_m_/T_n_) superlattices, where m and n denote the stacking sequence of SIO and STO, respectively (Supplementary Fig. 1). Figure 1b–d shows the low magnification high angle annular dark-field (HAADF)-scanning transmission electron microscopic (STEM), atomically resolved HAADF, and integrated differential phase contrast (iDPC) images for the I_3_/T_6_ superlattice, where a non-equivalent interface is constructed: one SrO layer at the perovskite interface while two SrO layers between TiO_2_ and IrO_2_ at the Ruddlesden–Popper interface. This unique interfacial structure naturally breaks the space-inversion symmetry in the SIO/STO superlattices.

### Dielectric and ME anomalies

**Fig. 2:**
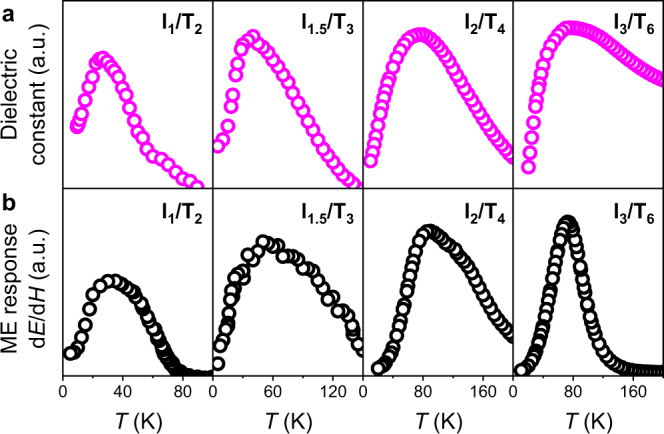
Dielectric and magnetoelectric (ME) anomalies in the Sr_2_IrO_4_/SrTiO_3_ superlattices. **a** Temperature-dependent dielectric constant measured along the *z-*direction for series of SIO/STO superlattices. **b** ME response as a function of temperature by the standard dynamic measurement. The maximum ME output in these superlattices occurs when a dielectric anomaly appears.

Dielectric properties were characterized in superlattices with different periods. Abnormally, a dielectric transition measured along the *z*-direction is observed in the I_m_/T_n_ superlattices (*m* from 1 to 3) from 24 to 77 K as shown in Fig. 2a, indicating the existence of a possible polarization. From the measurement of temperature-dependent magnetic moment (see Supplementary Fig. 2), all SIO/STO superlattices show antiferromagnetic behaviors^43^, implying their possible multiferroic nature by artificial design. A standard dynamic measurement (see Methods and Supplementary Fig. 3) was carried out to investigate the ME response as a function of temperature, which is an effective method to characterize the emergent phase transition^46^. There are peaks of the temperature-dependent ME output in these superlattices when the dielectric anomaly appears as shown in Fig. 2b. In order to further understand the anomalies of the dielectric and ME responses, the pyroelectric current was collected in I_3_/T_6_ as a function of temperature (see Methods and Supplementary Fig. 3). Spontaneous polarization also emerges, increases and then saturates near the dielectric anomaly as shown in Supplementary Fig. 4. While applying a magnetic field bias along the *z*-direction, the spontaneous polarization is suppressed up to 23% near this transition at ~77 K. This static measurement further confirms the existence of a direct ME effect.

### Ferroelectricity and interfacial DMI

**Fig. 3:**
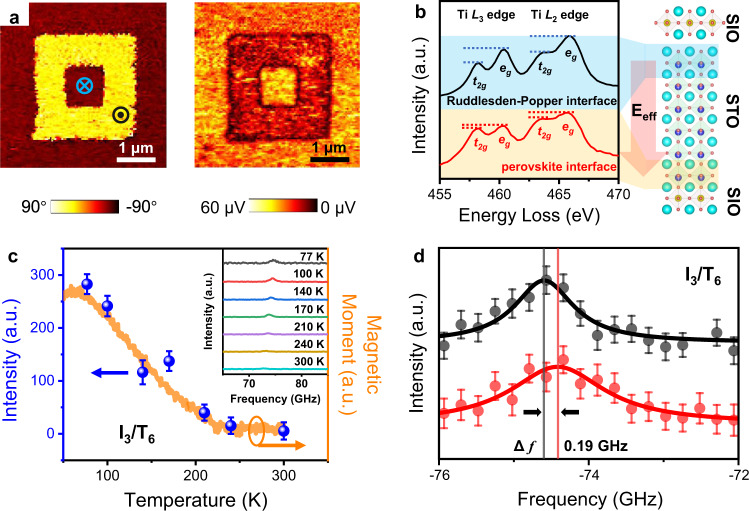
Ferroelectric switching and interfacial Dzyaloshinskii–Moriya interaction (DMI). **a** Out-of-plane PFM images of the I_3_/T_6_ superlattice at 3.7 K, the relative dark and bright contrasts in-phase (left panel) and amplitude (right panel) indicate upward and downward ferroelectric domains. **b** Electron energy loss spectroscopy (EELS) of Ti *L*_2,3_ for the I_3_/T_6_ superlattice corresponding to the detailed electronic structure. The intensity ratios of *e*_g_ to *t*_2g_ of the Ti *L*_2,3_ edges are reduced at the perovskite interface (red curve) which is different from that at the Ruddlesden–Popper interface (black curve). The distinct electronic structures between two interfaces could induce an effective electric field (**E**_eff_) in the STO. **c** Temperature-dependent resonant peak intensity of spin-wave for I_3_/T_6_ superlattice. When temperature increases, this resonant peak was dramatically suppressed as the decrease of magnetization (orange curve). Inset shows the temperature dependence of spin-wave spectra by BLS measurements. **d** BLS spectra for the I_3_/T_6_ superlattice. The difference of frequency (${\triangle f}$ = 0.19 GHz) between anti-Stokes (black curve) and Stokes (red curve) peaks demonstrates that the interfacial DMI exists in this superlattice. The error bars in (**c**) and (**d**) are standard errors of measurements.

To study the physical origin of the spontaneous polarization, temperature-dependent piezoresponse force microscopy (PFM) and atomically resolved electron energy loss spectroscopy (EELS) have been carried out in the I_3_/T_6_ superlattice (see Methods). The PFM phase and amplitude images in Fig. 3a show that there is a ferroelectric domain switching at 3.7 K under an up/down electric field. The coercive bias decreases when the temperature increases from 3.7 to 70 K and the ferroelectric switching disappears at 90 K as shown in Supplementary Fig. 5, consistent with the dielectric and pyroelectric measurements. The EELS exhibits a fingerprint of the occupancy of the Ti 3*d* orbitals, corresponding to the modulation of Ti oxidation state. In the I_3_/T_6_ superlattice, different electron occupations in *e*_g_ and *t*_2g_ orbitals^47^ have been verified from the fine structures of Ti *L*_2,3_ edges between the perovskite and Ruddlesden–Popper interfaces: Ti^4+^ at the Ruddlesden–Popper interface, but a mixed Ti^4+^ and Ti^3+^ at the perovskite interface as shown in Fig. 3b (details in Supplementary Note 1). Such different electronic structures manifest an asymmetric charge distribution, which provides an effective electric field (**E**_eff_) to the STO along the *z* direction^48^ and could lead to a ferroelectric polarization^44,45^. Thus, the **E**_eff_/spontaneous polarization and the asymmetric atomic structure between the perovskite and Ruddlesden–Popper interfaces provide a possible prerequisite for an interfacial DMI^27,36^.

In order to further evaluate the interfacial DMI, Brillouin light scattering (BLS) measurement has been performed, which is a well-established technique for the study of the interfacial DMI by characterizing spin waves with high sensitivity using the inelastic scattering of light (see “Methods”)^49^. As shown in Fig. 3c, resonant excitation of surface spin wave (magnon) is observed near 74 GHz (details in Supplementary Note 2). When temperature increases, this resonant peak is dramatically suppressed as the decrease of magnetization. Meanwhile, it is also observed that there is a non-reciprocal scattering for the opposite magnon wave vectors with a pronounced frequency shift of 0.19 GHz for this peak (Fig. 3d). The strength of interfacial DMI, $D$, is proportional to this frequency shift, $\triangle f$, between anti-Stokes and Stokes peaks in BLS spectra, i.e.^49^

1 $$D=\frac{{{{{{\rm{\pi }}}}}}\triangle f\,{M}_{s}}{2\gamma k}$$

where ${M}_{s}$ is the magnetic moment, $\gamma$ is the gyromagnetic ratio, and $k$ is the wave vector. This observed frequency difference ($\triangle f$) of 0.19 GHz suggests the existence of interfacial DMI (in-plane **D**_**int**_ vector) of this superlattice.

### ME phase transition

**Fig. 4:**
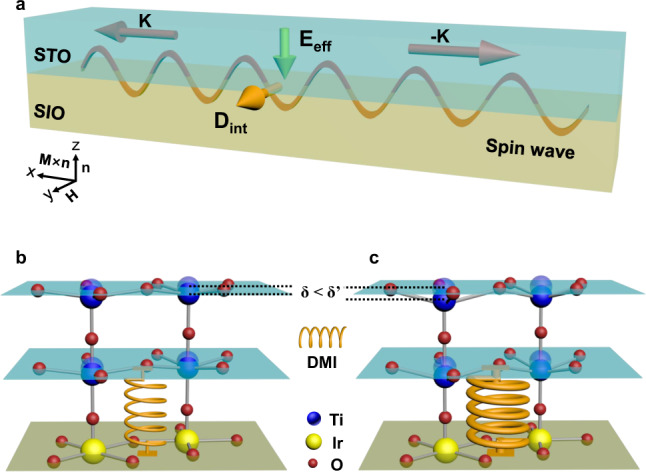
Origin of ME phase transition in the Sr_2_IrO_4_/SrTiO_3_ superlattices. **a** Schematic illustration of the in-plane **D**_**int**_ vector induced by the *z*-direction **E**_eff_ measured by the BLS. **K** and **−K** are the propagation directions of the spin-wave. **b**, **c** The microscopic scenario of the ME phase transition. The asymmetric-interface-induced polarization (effective electric field) can be illustrated by the atomic displacement (*δ* in (**b**) and *δ*’ in (**c**)). The effective electric-field-dependent DMI drives the ME phase transition, where the amplitude of DMI is schematically equivalent to the strength of spring (thin in (**b**) and thick in (**c**)). Guided by the theoretical model, the enhancement of the polarization (*δ* < *δ*’) and thus interfacial DMI could increase the temperature of the ME phase transition.

As schematically illustrated in Fig. 4a, the **D**_**int**_ vector induced by the out-of-plane **E**_eff_ is along the *xy*-plane. Therefore, the observed **E**_eff_ and **D**_**int**_ can be theoretically understood by the effective spin Hamiltonian^36,37^

2 $$H= -{J}_{{{{{\rm{eff}}}}}}\mathop{\sum} _{ < {{{{{\boldsymbol{i}}}}}},{{{{{\boldsymbol{j}}}}}} > }{{{{{{\bf{S}}}}}}}_{{{{{{\boldsymbol{i}}}}}}}\cdot {{{{{{\bf{S}}}}}}}_{{{{{{\boldsymbol{j}}}}}}}-{A}_{c}\mathop{\sum} _{{{{{{\boldsymbol{i}}}}}}}\left({{{{{{\bf{S}}}}}}}_{{{{{{\boldsymbol{i}}}}}}}^{{{{{{\boldsymbol{y}}}}}}}{{{{{{\bf{S}}}}}}}_{{{{{{\boldsymbol{i}}}}}}+\hat{{{{{{\boldsymbol{x}}}}}}}}^{{{{{{\boldsymbol{y}}}}}}}+{{{{{{\bf{S}}}}}}}_{{{{{{\boldsymbol{i}}}}}}}^{{{{{{\boldsymbol{x}}}}}}}{{{{{{\bf{S}}}}}}}_{{{{{{\boldsymbol{i}}}}}}+\hat{{{{{{\boldsymbol{y}}}}}}}}^{{{{{{\boldsymbol{x}}}}}}}\right)\\ -D\mathop{\sum}\limits_{{{{{{\boldsymbol{i}}}}}}}\left[\hat{{{{{{\bf{y}}}}}}}\cdot \left({{{{{{\bf{S}}}}}}}_{{{{{{\boldsymbol{i}}}}}}}\times {{{{{{\bf{S}}}}}}}_{{{{{{\boldsymbol{i}}}}}}+\hat{{{{{{\boldsymbol{x}}}}}}}}\right)-\hat{{{{{{\boldsymbol{x}}}}}}}\cdot \left({{{{{{\bf{S}}}}}}}_{{{{{{\boldsymbol{i}}}}}}}\times {{{{{{\bf{S}}}}}}}_{{{{{{\boldsymbol{i}}}}}}+\hat{{{{{{\boldsymbol{y}}}}}}}}\right)\right]$$

where ${{{{{{\bf{S}}}}}}}_{{{{{{\boldsymbol{i}}}}}}}$ and ${{{{{{\bf{S}}}}}}}_{{{{{{\boldsymbol{j}}}}}}}$ are the magnetic moment at the site ***i*** and ***j***, $\hat{{{{{{\boldsymbol{x}}}}}}}$ and $\hat{{{{{{\boldsymbol{y}}}}}}}$ are unit vectors. The effective Heisenberg coupling in the first term of Eq. (2) is ${J}_{{{{{\rm{eff}}}}}}=\widetilde{J}{\cos }2\delta$, the compass anisotropy in the second term is ${A}_{c}=\widetilde{J}\left(1-{\cos }2\delta \right)$ and the interfacial DMI in the third term is $D=\widetilde{J}{\sin }2\delta$. Here, ${\tan }\delta =\frac{\lambda }{t}$, and $\widetilde{J}$ is proportional to $\sqrt{{t}^{2}+{\lambda }^{2}}$, where $\lambda$ and $t$ are the coefficient of spin–orbit coupling and the hopping parameters, respectively. The origin of the interfacial DMI and compass anisotropy lies in the strength of spin–orbit coupling ($\lambda$). In general materials, $\lambda \ll t$, we can deduce that ${\tan }\delta \simeq \delta =\frac{\lambda }{t}$ . Therefore, the interfacial DMI coefficient $D=\widetilde{J}{\sin }2\delta \simeq 2\delta \widetilde{J}\simeq 2\lambda$, which is proportional to the ferroelectric polarization due to $\lambda \propto$ **E**_eff_^50^. Furthermore, the role of this interfacial DMI on the phase transition of temperature-dependent ME response can be estimated as^51^ ${k}_{{{{{{\rm{B}}}}}}}{T}_{c} \sim {J}_{eff}\,{({{{{\mathrm{ln}}}}}(\frac{\beta }{\lambda }))}^{-1}$, where the ${T}_{c}$ is the critical transition temperature, $\beta$ is a phenomenological parameter (Supplementary Note 3). We define this transition as the ME phase transition. The ${T}_{c}$ increases with $\lambda$, namely, proportional to the $D$ or **E**_eff_/polarization. Figure 4b shows the microscopic scenario of the ME phase transition driven by the interfacial DMI (denoted schematically by the Spring). ${T}_{c}$ is proportional to $D$ or spontaneous polarization. Therefore, artificial engineering by introducing additional contribution of polarization for enhancing the interfacial DMI (a thicker Spring) may be effective to achieve a ME phase transition at high temperature as shown in Fig. 4c.

**Fig. 5:**
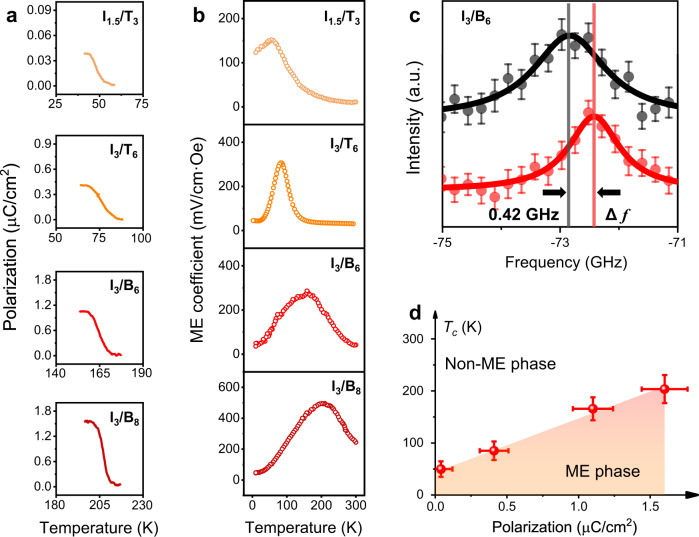
ME phase transition by the engineering of interfacial DMI. **a**, **b** Temperature-dependent spontaneous polarization and ME response for I_1.5_/T_3_, I_3_/T_6_, I_3_/B_6_, and I_3_/B_8_ superlattices, respectively. The temperature of ME phase transition could be increased from 46 to 203 K by the enhancement of polarization (from 0.04 to 1.62 µC/cm^2^). The polarization was obtained by the integration of pyroelectric current. **c** BLS spectra of the I_3_/B_6_ superlattice. The difference of frequency (${\triangle f}$ = 0.42 GHz) is twice larger than the one in I_3_/T_6_ superlattice (${\triangle f}$ = 0.19 GHz), demonstrating the enhancement of interfacial DMI due to the increased polarization of the I_3_/B_6_ superlattice. **d** The ME phase diagram. The error bars in (**c**) and (**d**) are standard errors of measurements.

For this purpose, BaTiO_3_ (BTO) with intrinsic polarization was introduced into the superlattices to further increase the **E**_eff_ and polarization. Figure 5a shows the temperature-dependent polarizations in SIO/STO and SIO/BTO superlattices. Spontaneous polarization of 0.04, 0.41, 1.12, and 1.62 µC/cm^2^ are obtained in the I_1.5_/T_3_, I_3_/T_6_, I_3_/B_6_, and I_3_/B_8_ superlattices. This enhanced polarization directly leads to the ME phase transition at high temperatures as shown in Fig. 5b, which is benefitted from the enhancement of the interfacial DMI as seen in Fig. 5c (doubled frequency difference ($\triangle f$) of 0.42 GHz in the non-reciprocal scattering) and details in Supplementary Note 4. Such an enhanced interfacial DMI can be interpreted by the asymmetry of lattice distortions at both interfaces and the observed polar distortions in SIO/BTO superlattices (Supplementary Note 5). It should be noted that other possibilities may also contribute to the DMI, such as structural inhomogeneities. But according to the vector relationship analyzed by the theoretical and experimental results, the asymmetric atomic structures between two interfaces should be the main origin of this enhanced interfacial DMI. The ${T}_{c}$ are about 46, 77, 166, and 203 K for I_1.5_/T_3_, I_3_/T_6_, I_3_/B_6_, and I_3_/B_8_ superlattices respectively. The relation between ${T}_{c}$ and the polarization is plotted in the diagram (Fig. 5d). It is also notable that a pronounced ME coefficient of ~495 mV/cm·Oe without the application of magnetic bias is observed near the ${T}_{c}$ of I_3_/B_8_ superlattice by engineering the polarization. Apart from this direct ME effect, polarization or effective-field control of the interfacial DMI (Fig. 3d and Fig. 5c) indicates a potential converse ME effect in the superlattices. This mutual control of polarization and magnetism provides the versatile design of ME functionalities^33^.

In summary, an emergent ME phase transition has been achieved by an artificial design of Ruddlesden–Popper antiferromagnetic and perovskite paraelectric/ferroelectric superlattices. The phase transition temperature can be manipulated from 46 to 203 K via the engineering of the interfacial DMI, accompanying a pronounced ME response. This controllable ME effect implies its great potential for developing functionalities in integrated magnetoelectronic superlattices. Furthermore, atomic construction of the superstructures in layered- (e.g., A_2_BO_4_ and A_3_B_2_O_7_) and traditional-perovskite (ABO_3_) oxides naturally provides a framework toward non-centrosymmetric systems with strong electron correlation for exploring emergent quantum phenomena.

## Methods

### Sample growth and structural characterizations

SIO/STO and SIO/BTO superlattices were fabricated by Laser-Molecular beam epitaxy (TSST) on etched STO (001) substrates with TiO_2_-termination. For deposition of all the superlattices, a KrF excimer laser with a wavelength of 248 nm and an energy density of ~1 J/cm^2^ was used. The films were grown at 800 °C under an oxygen pressure of 0.02 mbar. The repetition rate was 2 Hz. The same condition was used to grow paramagnetic LaNiO_3_ as the bottom electrode. After deposition, the films were slowly cooled (5 °C/min) under an oxygen pressure of 100 mbar. The layer-by-layer growth mode was monitored by reflection high-energy electron diffraction oscillations (Supplementary Fig. 1). All the superlattices were controlled with the same thickness. Synchrotron X-ray diffraction measurements were carried out at room temperature at beamline 1W1A of the Beijing Synchrotron Radiation Facility.

### Piezoresponse force microscopy (PFM)

The temperature-dependent PFM and piezoresponse phase hysteresis were carried out on Attocube DRY1000 with the temperature range from 3.7 to 90 K. A PtIr5-coated tip (NanoSensors) with a radius of <25 nm was used. The amplitude and frequency of the AC input were 2*V*_pp_ and 22 kHz, respectively. The upward and downward ferroelectric domains could be written by applying a positive (+8 V) and negative (−8 V) bias on the tip^52–55^.

### Scanning transmission electron microscope (STEM) and electron energy loss spectroscopy (EELS)

The atomic-resolution STEM images were recorded using an aberration-corrected Titan Themis G2 microscope at 300 kV with a beam current of 50 pA. The convergence semi-angle of the incident electron probe was approximately 30 mrad, and the HAADF images were collected over a detector angle range of 39–200 mrad. The atomically resolved iDPC image along the pseudo-cubic [010] zone axis was also performed at room temperature. The EELS was acquired by Nion UltraSTEM^TM^ 200 microscope with aberration corrector and monochromator operating at 60 kV with a beam current of 20 pA. The probe convergence semi-angle was 35 mrad and the collection semi-angle was 24.9 mrad. The electronic occupation state of titanium with the spatial resolution was compared by *L*_2,3_ edges as in the previous work^56–58^. All the STEM and EELS are collected at room temperature. The measured periods of samples are (SIO)_3_/(STO)_6_ and (SIO)_3_/(BTO)_6_ superlattices with the repeated units of 20. The STO or BTO layer is the top layer.

We acquired single 20 × 70 pixels EELS mapping from a region of 4 × 14 nm containing two periods of SIO/STO superlattice. The dwell time was 300 ms/pixel, the dispersion was 0.1663 eV/ch. The energy solution was estimated at ~0.87 eV. The data was smoothed by the Gaussian-weighted moving average filter with a window length of 2 channels and a variance of 7 channels. The EELS spectra were taken on the same single EELS mapping and are spatially averaged over the regions indicated by the blue lines in Supplementary Fig. 6a.

### Dielectric, magnetic, and pyroelectric current measurements

The temperature-dependent dielectric constant was measured by Precision LCR Meter (Keysight E2980 AL) along the *z*-direction equipped with cryostats (PPMS DynaCool; Quantum Design).

The temperature-dependent magnetic moment was obtained by a 5 T SQUID magnetometer (Quantum Design). The field (1000 Oe) was applied along the in-plane direction of the superlattices to detect the magnetic moment after zero-field cooling, which is to detect the spin-canted antiferromagnetic structure as a function of temperature^59^.

All the pyroelectric current was measured by an electrometer (Keithley 6517B) along the *z*-direction, in which an external DC magnetic field could be applied. Before the pyroelectric measurement, the samples were cooled down by a Cryogen-free Superconducting Magnet System (Oxford Instruments, TeslatronPT) under the poling procedure with the cooling field of 4.5 kV/cm, then the pyroelectric current was detected under zero electric field within the warming process of 1 K/min. The spontaneous polarization with a DC magnetic field (static ME response) was obtained by the integration of the pyroelectric current^60–62^.

### Dynamic measurement of the ME response

The temperature-dependent ME response was measured in a Cryogen-free Superconducting Magnet System (Oxford Instruments, TeslatronPT) using a dynamic technique. AC current was applied to a solenoid to generate an alternating magnetic field (~2.5 Oe) by Keithley 6221 AC source, and the ME-induced AC voltage on the samples was collected by a lock-in amplifier (Stanford Research SR830). Coaxial cables were used in all the measurements. The detected voltage signals in the superlattices are nearly 2 orders larger than the electromagnetic induction and other noise signals, thus the ME voltage signal can be attained by sensitive detection^63–65^.

### Brillouin light scattering (BLS)

The laser source is a single-mode-stabilized 532 nm polarized light with a power of 30 mW. Liquid nitrogen was used in cryo-temperature equipment for cooling and temperature control, and the temperature can be controlled in the range of 77–300  K. During the test, the temperature fluctuation range was within 5 K. The laser is incident into the transparent window of the cryogenic equipment at a fixed angle and is focused on the samples. The scattered light collected by an objective lens passes through a multi-pass tandem Fabry–Pérot interferometer (TFP-2) to be frequency-analysed^66,67^. The test time of each BLS data is about 2 h.

## Supplementary information

Supplementary Information

## Data Availability

The authors declare that the data supporting the findings of this study are available from the corresponding author upon reasonable request.
