# Supplementary material for: Magnetoelectric phase transition driven by interfacial-engineered Dzyaloshinskii-Moriya interaction
Source: Nat Commun. 2021 Sep 15;12:5453. doi: 10.1038/s41467-021-25759-1 (PMC8443571; doi:10.1038/s41467-021-25759-1)
Supplement: Supplementary file 1 — Supplementary Information [file 41467_2021_25759_MOESM1_ESM.pdf]

Supplementary Information

Magnetoelectric Phase Transition Driven by Interfacial-engineered Dzyaloshinskii-Moriya Interaction

Xin Liu[1#], Wenjie Song[2#], Mei Wu[3,4#], Yuben Yang[1#], Ying Yang[1], Peipei Lu[5,6], Yinhua Tian[2], Yuanwei Sun[3,4], Jingdi Lu[1], Jing Wang[7,8], Dayu Yan[5], Youguo Shi[5], Nian Xiang Sun[9], Young Sun[5,6], Peng Gao[3,4,10*], Ka Shen[1], Guozhi Chai[2], Supeng Kou[1], Ce-Wen Nan[7*] and Jinxing Zhang[1*]

[1]Department of Physics, Beijing Normal University, 100875 Beijing, PR China

[2]Key Laboratory for Magnetism and Magnetic Materials of the Ministry of Education, Lanzhou University, 730000 Lanzhou, PR China

[3]International Center for Quantum Materials, Peking University, 100871 Beijing, PR China

[4]Electron Microscopy Laboratory, School of Physics, Peking University, 100871 Beijing, PR China

[5]Beijing National Laboratory for Condensed Matter Physics, Institute of Physics, Chinese Academy of Sciences, 100190 Beijing, PR China

[6]School of Physical Science, University of Chinese Academy of Sciences, 100190 Beijing, PR China

[7]School of Materials Science and Engineering, Tsinghua University, 100084 Beijing, PR China

[8]Advanced Research Institute of Multidisciplinary Science, Beijing Institute of Technology, 100081 Beijing, PR China.

[9]Department of Electrical and Computer Engineering, Northeastern University, 02115 Boston, Massachusetts, USA

[10]Collaborative Innovation Centre of Quantum Matter, 100871 Beijing, PR China

# These authors contributed equally: Xin Liu, Wenjie Song, Mei Wu and Yuben Yang

*email: p-gao@pku.edu.cn; cwnan@mail.tsinghua.edu.cn; jxzhang@bnu.edu.cn

Supplementary Notes and Figures

Section I: Sample growth, structural and magnetic characterizations.

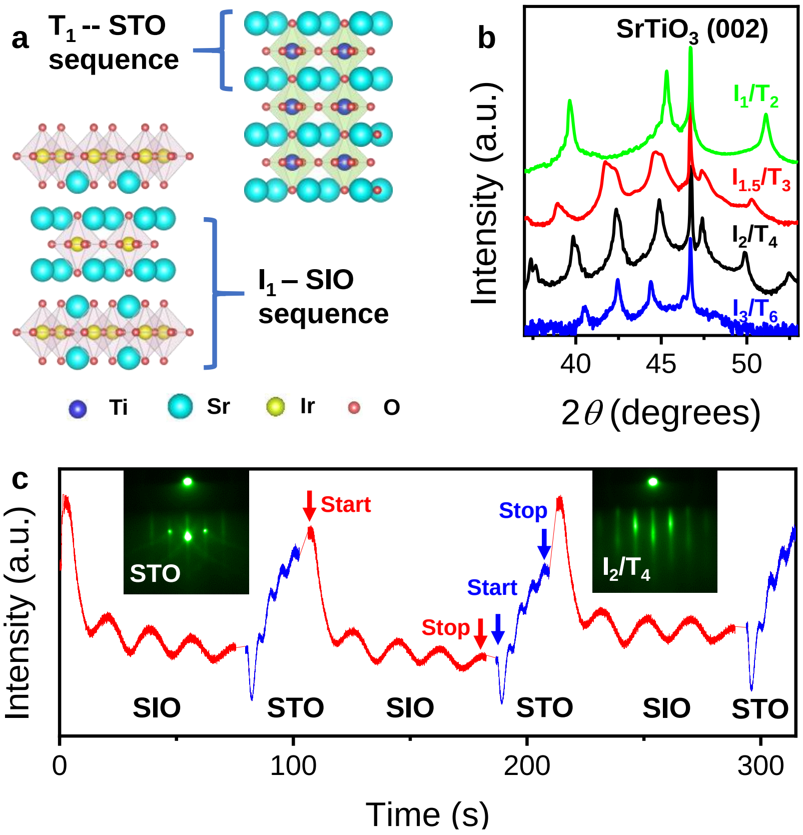

Supplementary Figure 1 | High-quality epitaxial $Sr_2IrO_4/SrTiO_3$ (SIO/STO) superlattices. a, Schematic of the stacking sequence of SIO and STO in SIO/STO superlattices. **b**, θ-2θ X-ray diffractograms of SIO/STO superlattices on STO substrate, confirming the periodicity and high crystallinity. **c**, Reflection high-energy electron diffraction (RHEED) oscillations for layer-by-layer growth of the $(SIO)_2/(STO)_4$ (I_2/T_4) superlattice. Inset shows the RHEED diffraction patterns of STO substrate and I_2/T_4 superlattice, indicating a two-dimensional character. For the growth of SIO layers, each oscillation demonstrates the deposition of SrO-IrO_2-SrO sequence, due to the identical stoichiometric layers shifted by lattice translations[1]. For the growth of STO layers on the SrO-terminated SIO, each oscillation demonstrates the deposition of TiO_2-SrO sequence. After the alternative growth, asymmetric interfaces were spontaneously constructed in the superlattices.

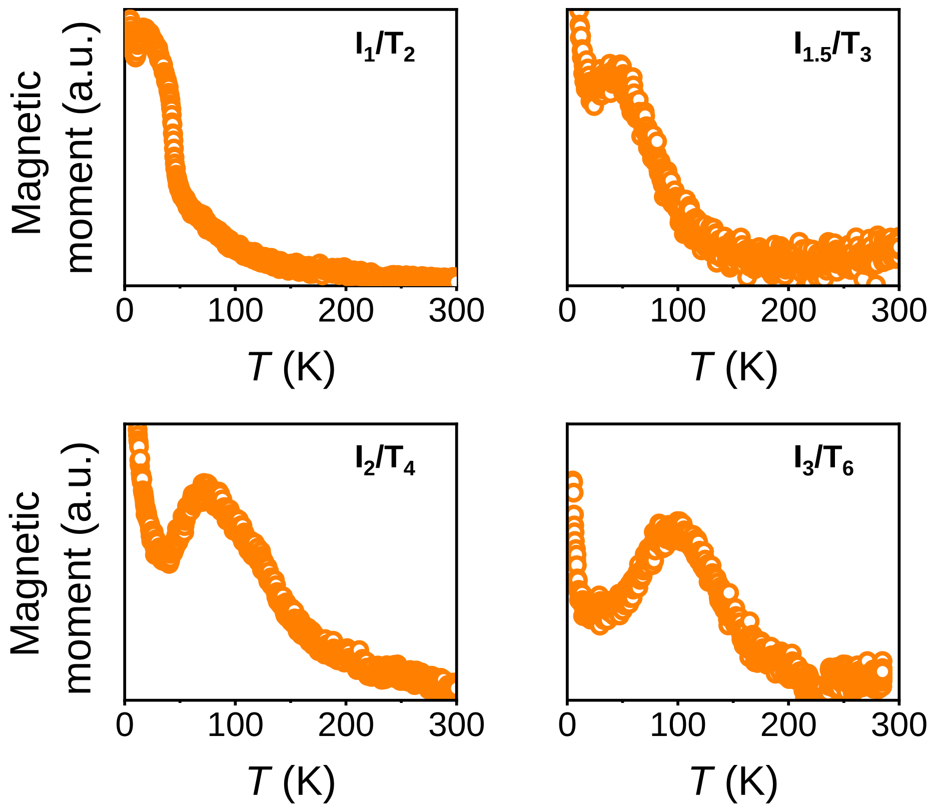

Supplementary Figure 2 | Temperature-dependent magnetic moment measured within the *xy*-plane for series of SIO/STO superlattices.

Section II: Dynamic and static measurements of the magnetoelectric (ME) response.

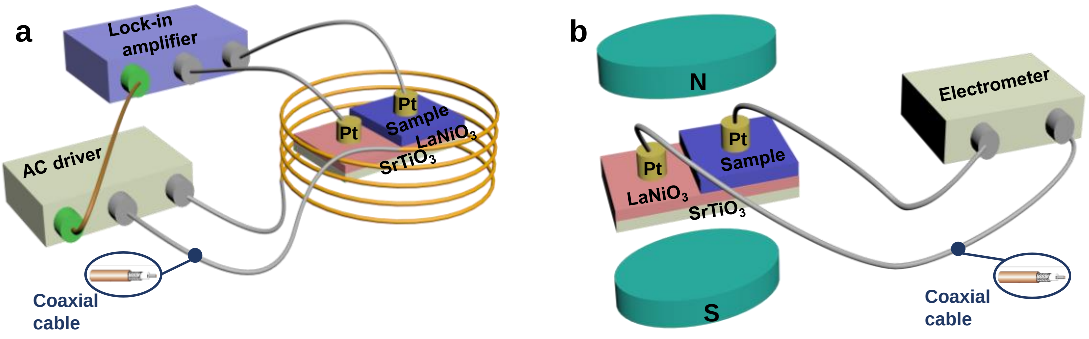

Supplementary Figure 3 | Schematic illustration of dynamic and static measurements of ME response. a, AC current was applied to a solenoid to generate an alternating magnetic field (~2.5 Oe) perpendicular to the sample by AC driver, and the ME-induced AC voltage on the sample was collected by a lock-in amplifier. **b**, Current was collected based on a pyroelectric measurement by the electrometer during the heating process. DC magnetic field could be applied perpendicularly to the sample for static ME response. Coaxial cables were used in all measurements.

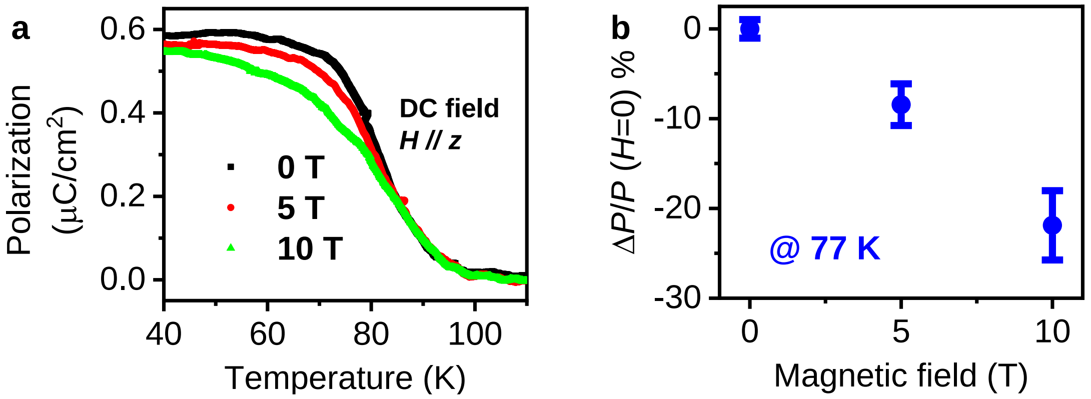

Supplementary Figure 4 | Static measurement of the direct ME effect. a, Temperature dependence of spontaneous polarization under external DC magnetic field along the *z* axis. The polarization was obtained by integration of pyroelectric current. The suppression of polarization under the DC magnetic field near the transition temperature was summarized in **(b)**. The error bars are standard error of measurements.

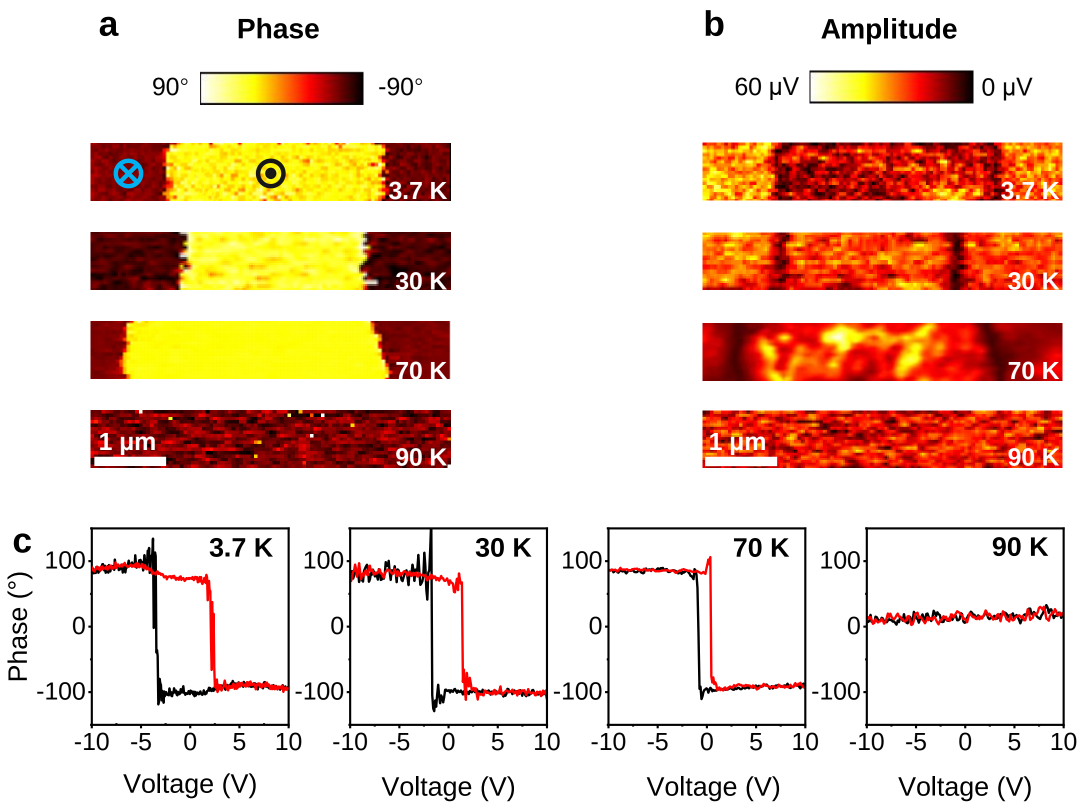

Supplementary Figure 5 | Temperature-dependent Piezoresponse force microscopy (PFM) characterizations. a,b, Temperature-dependent PFM images of the I_3/T_6 superlattice. The relative dark and bright areas in phase **(a)** and amplitude **(b)** images indicate upward and downward ferroelectric domains. The ferroelectric switching disappears at 90 K. **c**, Temperature-dependent piezoresponse phase hysteresis of the I_3/T_6 superlattice. The coercive bias decreases when temperature increases from 3.7 K to 70 K and disappears at 90 K.

Section III: Analysis of charge imbalance, lattice structure and the interfacial Dzyaloshinskii-Moriya interaction (DMI).

Supplementary Note 1: The origin of the charge imbalance.

Electron energy loss spectroscopy (EELS) of Ti-*L* and O-*K* edges are very sensitive to Ti valence and oxygen vacancy. Therefore, in order to clearly compare the oxygen environments between the Ruddlesden-Popper and perovskite interfaces, we simultaneously recorded atomically resolved EELS spectra of the Ti-*L* and O-*K* edges corresponding to each interface as shown in Supplementary Fig. 6a-6e. The intensity ratio of e_g to t_{2g} of the Ti-$L_{2,3}$ edges are reduced at the perovskite interface (pink and orange curves) compared to the ones at the Ruddlesden-Popper interface (black and blue curves) as shown in Supplementary Fig. 6d, illustrating the mixed-valence of Ti cations at the perovskite interface. This result is well consistent with the observation in Fig. 3b. What's more, reduced energy splitting of e_g and t_{2g} is also observed in the perovskite interface, further confirming the reduced valence state (Supplementary Fig. 7a). However, the extracted O-*K* edge appears approximately the same fine structure between the two interfaces (Supplementary Fig. 6e), indicating the identical oxygen chemical environment without a distinguishable difference in oxygen concentration. Based on the above experiments and analysis, although there are Ti^{4+} at one interface and mixed Ti^{4+}/Ti^{3+} at the other interface, we observe no distinguishable difference between the states of oxygen at the two interfaces. Therefore, most likely, the reason for the appearance of the Ti^{3+} ion at the perovskite interface is not oxygen vacancies.

Then, in order to clarify the reason behind, we further investigated the oxidation state of Ir. Since the STEM-EELS is typically used to detect energy loss lower than 3 keV and not suitable for Ir-*L* edge, instead we carried out synchrotron X-ray absorption spectroscopy (XAS) at the BL39XU of Spring-8 Japan to collect the spectra near L_3 edge of Ir in the $(Sr_2IrO_4)_3/(SrTiO_3)_6$ superlattice and pure Sr_2IrO_4 thin film for comparison. The Ir L_3 edge XAS spectra were collected by standard helicity reversal

technique with a grazing incidence geometry (5.5° incidence angle). The partial fluorescence yield (PFY) mode was conducted, where the emissions were collected and energy-analyzed by a four-element silicon drift detector (Sirius 4, SGX Sensortech Inc.), respectively. As shown in Supplementary Fig. 8, the XAS peak position shifts to higher energy in the $(Sr_2IrO_4)_3/(SrTiO_3)_6$ superlattice (inset Supplementary Fig. 8), compared to that in pure Sr_2IrO_4 thin film. This shift of XAS spectra implies that the valence of Ir increases in the superlattice compared to that in pure Sr_2IrO_4 thin film. Therefore, the appearance of the Ti^{3+} near the interface is possibly due to the change of Ir oxidation state.

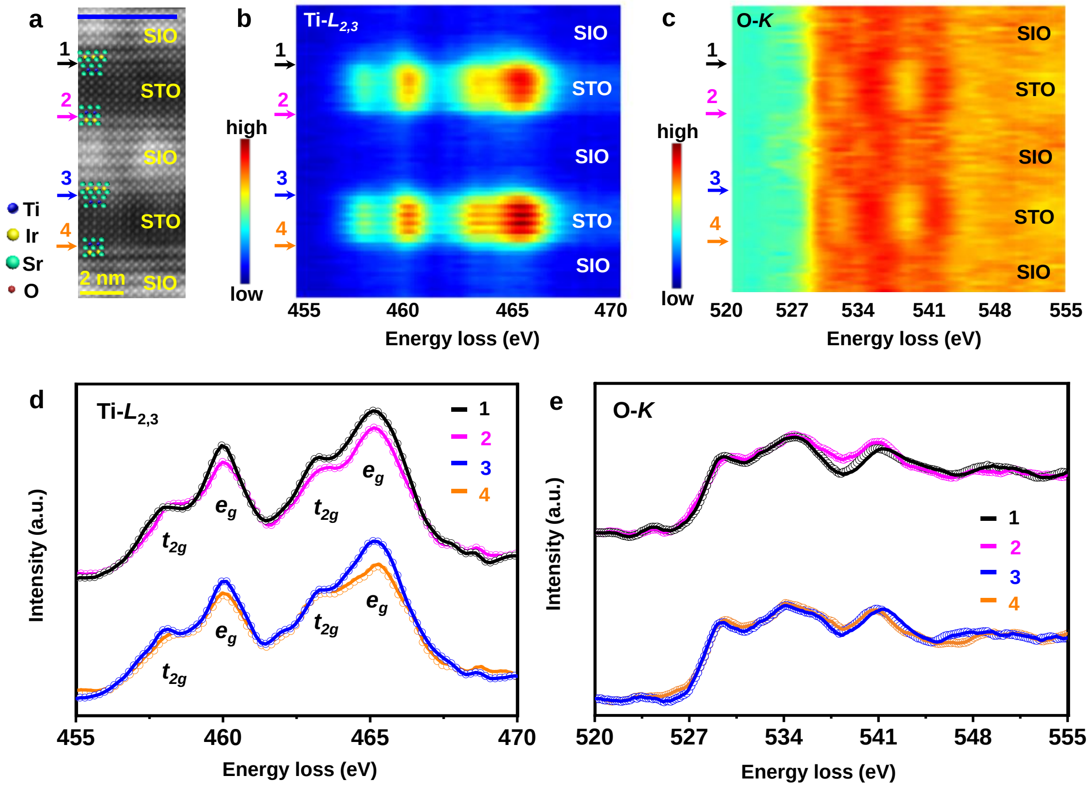

Supplementary Figure 6 | a, High angle annular dark field (HAADF)-scanning transmission electron microscopy (STEM) image of the $(Sr_2IrO_4)_3/(SrTiO_3)_6$ (SIO_3/STO_6) superlattice for the EELS acquirement. **b,c,** EELS mapping of Ti-$L_{2,3}$ **(b)** and O-K edges **(c)** across the SIO_3/STO_6 superlattice. **d,e,** A comparison for EELS of Ti-$L_{2,3}$ **(d)** and O-K edge as the arrows labeled in **(a)**.

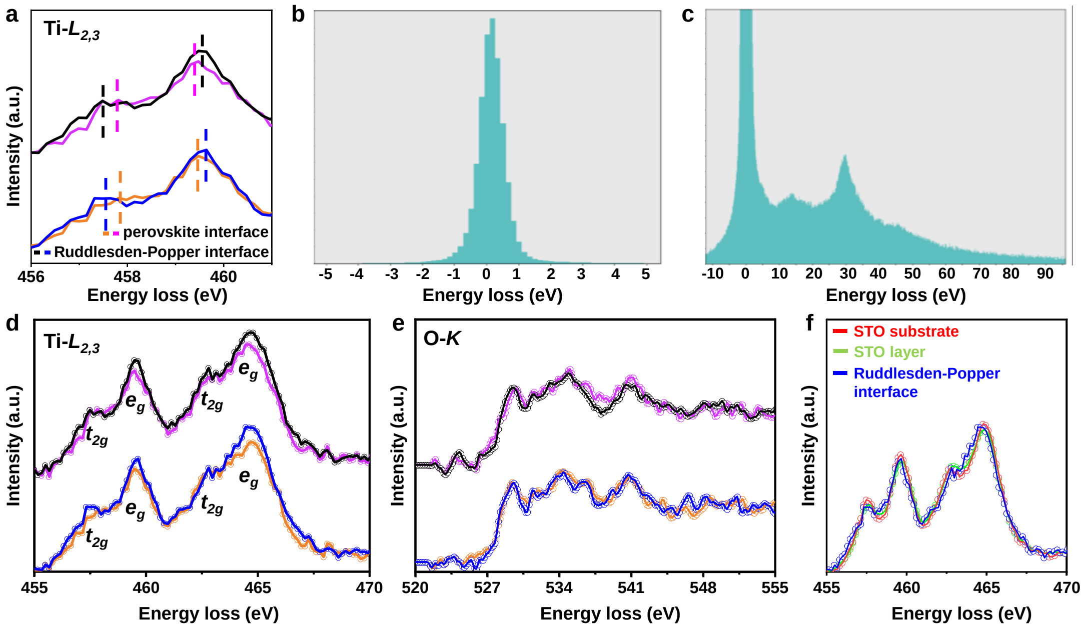

Supplementary Figure 7 | a, Enlarged Ti-*L* edge for different interfaces, indicating small t_{2g}, e_g splitting in the perovskite interface. **b**, The zero-loss peak with energy resolution ~0.87eV. **c**, EELS spectrum acquired in the SIO/STO superlattice ranged from -10 eV to 100 eV, indicating sample thickness is ~23.7 nm. **d,e**, A comparison for raw EELS of Ti-$L_{2,3}$ **(d)** and O-*K* edge **(e)** between the perovskite and Ruddlesden-Popper interfaces in two different regions as labeled in **(a)**. The results are consistent with the conclusion in Supplementary Fig. 6. **f,** A comparison for raw EELS of Ti-$L_{2,3}$ in STO substrate, interior STO layer and Ruddlesden-Popper interface illustrating nearly identical fine feature.

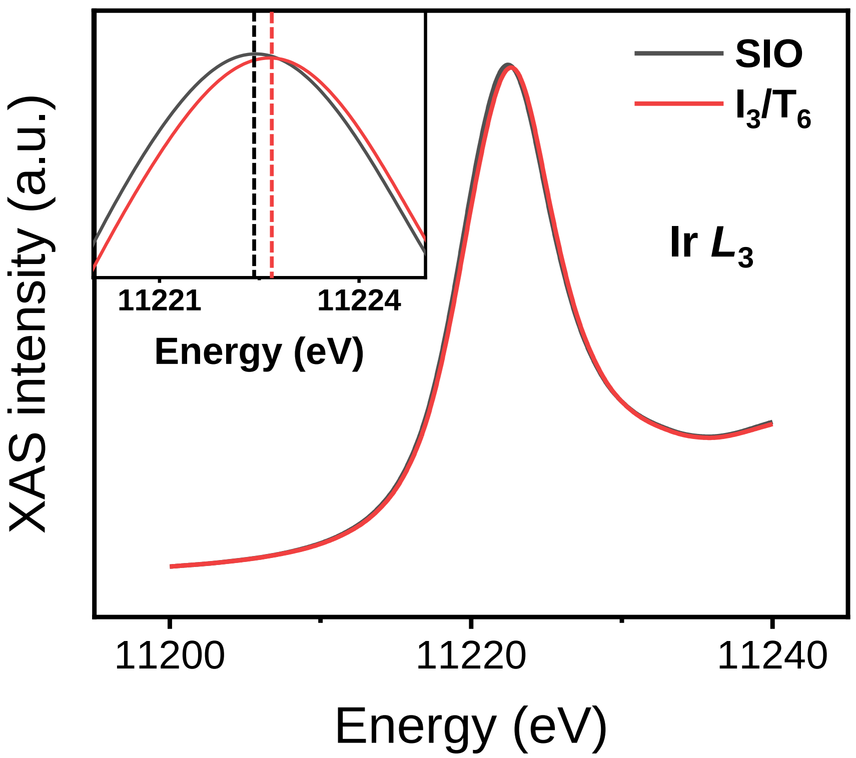

Supplementary Figure 8 | XAS spectra of the Ir-L_3 edge on I_3/T_6 superlattice and pure SIO thin film. The peak of the L_3 edge shifts to higher energy in I_3/T_6 superlattice (inset), indicating an enhanced Ir oxidation.

Section IV: Interfacial DMI and ME phase transition.

Supplemental Note 2: Non-reciprocity by BLS measurement.

Spin waves are collective magnetic excitations which flow strongly in the direction of $\mathbf{M} \times \mathbf{n}$, where **M** and **n** represent equilibrium magnetization under external magnetic field and normal vector of the interface, respectively[2]. In the BLS measurement, external magnetic field is along y direction (**H** ~1000 Oe). Thus, the spin wave propagates within the xz-plane. The propagation directions of the spin wave (**K** and **-K**) are perpendicular to the external magnetic field. The in-plane $\mathbf{D_{int}}$ vector driven by the perpendicular E_{eff} is confirmed by a frequency difference between the spin waves with **K** and **-K**, i.e., $\Delta f = f(K) - f(-K)$, which is measured by BLS[3].

Temperature-dependent BLS were carried out on the I_3/T_6 superlattice, a signal near 74 GHz was observed, when increasing the temperature, this peak was dramatically suppressed as shown in Fig. 3c. The temperature-dependent intensity of the signal has a strong correlation with the result in Supplementary Fig. 2, indicating that the observed signal is from surface spin wave[4].

Supplemental Note 3: Theoretical analysis for ME phase transition.

The effective spin Hamiltonian is expressed as[5]:

$$H = -J_{eff} \sum_{<i,j>} \mathbf{S}_i \cdot \mathbf{S}_j - A_c \sum_i \left(\mathbf{S}_i^y \mathbf{S}_{i+\hat{x}}^y + \mathbf{S}_i^x \mathbf{S}_{i+\hat{y}}^x \right)$$

$$-D \sum_i [\hat{\mathbf{y}} \cdot (\mathbf{S}_i \times \mathbf{S}_{i+\hat{x}}) - \hat{\mathbf{x}} \cdot (\mathbf{S}_i \times \mathbf{S}_{i+\hat{y}})] \quad (1)$$

where $\mathbf{S}_i$ and $\mathbf{S}_j$ are the magnetic moment at the site ***i*** and ***j***, $\hat{\boldsymbol{x}}$ and $\hat{\boldsymbol{y}}$ are unit vectors. The effective Heisenberg coupling is $J_{eff} = \tilde{J} cos2\delta$, the compass anisotropy is $A_c = \tilde{J}(1 - cos2\delta)$ and the interfacial DMI is $D = \tilde{J} sin2\delta$. Here, $tan\delta = \frac{\lambda}{t}$, and $\tilde{J}$ is proportional to $\sqrt{t^2 + \lambda^2}$, where λ and t are the coefficient of spin-orbit coupling and the hopping parameters, respectively. The DMI coefficient is proportional to the ferroelectric polarization due to $\lambda \propto \mathbf{E}_{\mathrm{eff}}$ according to the Hamiltonian of spin-orbit coupling: $H \sim \frac{\mu_B (E \times p) \cdot \sigma}{mc^2}$, where μ_B is the Bohr magneton, E is the electric field, p is the momentum of the electron, σ is the vector of the Pauli spin matrices, m is the mass of the electron and c is the speed of light.

Furthermore, the role of interfacial DMI on the temperature-dependent ME response is investigated based on symmetry analysis from Mermin-Wagner theorem[6]. *SU*(2) symmetry can be broken by the spin-orbit coupling, λ. For the case with $\lambda = 0$, the system becomes the isotropic Heisenberg model and the *SU*(2) symmetry persist the invariance, the critical temperature of order-disorder phase transition must be zero; for the case with $\lambda \neq 0$, the *SU*(2) symmetry is broken slightly and the critical temperature of order-disorder phase transition becomes finite. Namely, considering the polarization or interfacial DMI in the superlattices, a finite temperature for the phase transition can be driven. For further studying the transition temperature, we may have the free energy in quasi-2D magnetic systems[7]: $\Delta F = 4\pi J_{eff} - k_B T_c \ln(\frac{\beta}{\lambda})$, where β is a phenomenological parameter. The critical temperature (T_c) can be estimated as[8]: $k_B T_c \sim J_{eff} (\ln(\frac{\beta}{\lambda}))^{-1}$. Therefore, the transition temperature increases with λ, namely,

the transition temperature is proportional to the D or polarization ($T_c \propto \lambda \propto D$).

Section V: Engineering of Interfacial DMI and ME coefficient.

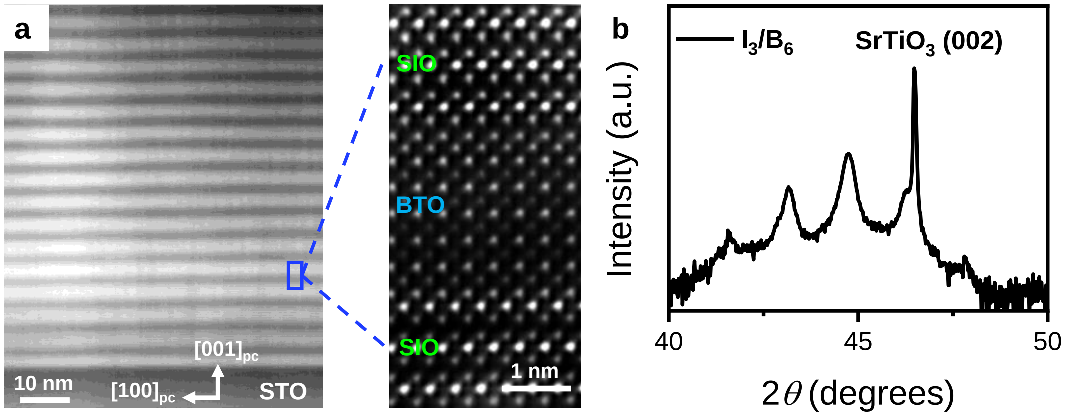

Supplementary Figure 9 | High-quality epitaxial SIO/BTO superlattices. a, High angle annular dark field (HAADF)-STEM images taken along the STO [010] zone axis, showing the I_3/B_6 superlattice with non-equivalent interfaces. **b**, θ-2θ X-ray diffractograms of the I_3/B_6 superlattice on STO substrate.

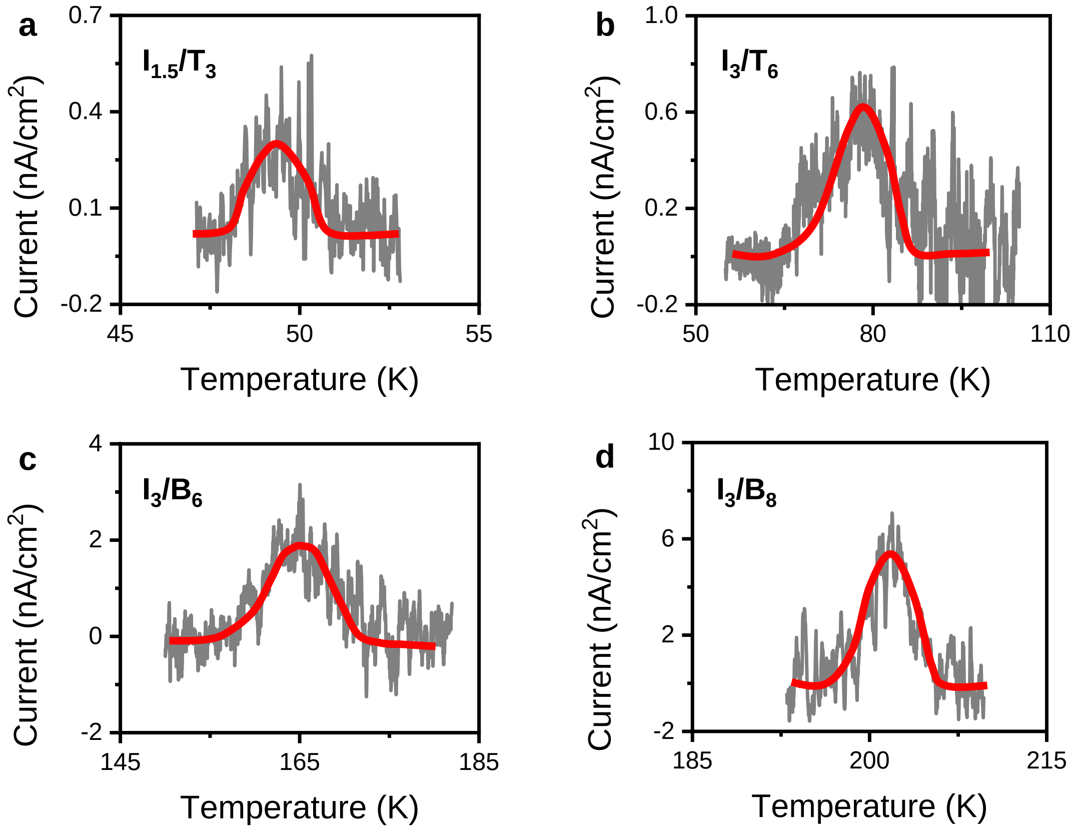

Supplementary Figure 10 | Pyroelectric current for series of superlattices. a,b,c,d, Temperature-dependent pyroelectric current measurements for $I_{1.5}/T_3$, I_3/T_6, I_3/B_6 and I_3/B_8 respectively. After poling procedure with the cooling field of 4.5 kV/cm, the pyroelectric current was collected under zero electric field with the warming process of 1 K/min.

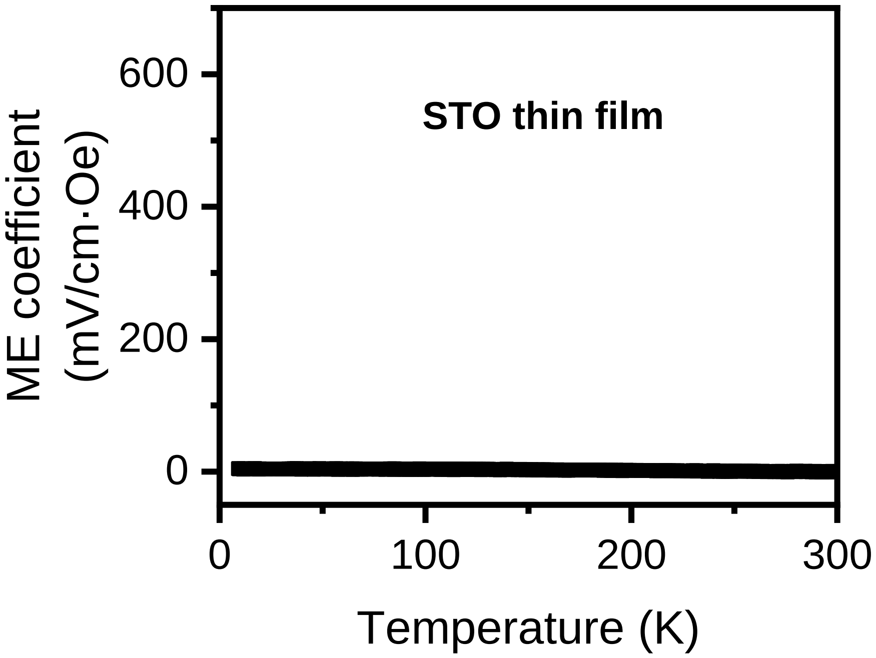

Supplementary Figure 11 | ME measurements on the STO thin film under the same conditions as a reference test. No ME phase transition was observed.

Supplemental Note 4: Relation between the transition temperature and DMI.

In order to clarify this relation between T_c and D, we estimate the interfacial DMI by conducting the BLS measurement. According to the equation[9]:

$$D = \frac{\pi\,\Delta f\, M_s}{2\gamma k} \tag{2}$$

where D is the strength of the interfacial DMI, M_s is magnetic moment, γ is gyromagnetic ratio, k is wave vector, and Δf is the frequency shift between anti-Stokes and Stokes peaks in BLS spectra. With the same M_s (~5 emu/cc), γ (2.8 GHz/kOe) and k ($\frac{4\pi}{\lambda} sin\frac{2\pi}{45}$, $\lambda = 532$ nm), doubled interfacial DMI (~34.8 μJ/m^2) was obtained in I_3/B_6 superlattice, where doubled frequency difference of 0.42 GHz was observed compared to the I_3/T_6 superlattice (Δf = 0.19 GHz, ~16.9 μJ/m^2). As shown in Fig. 5b, the T_c is indeed enhanced to 166 K.

Section VI: Analysis of the lattice structure and the interfacial Dzyaloshinskii-Moriya interaction (DMI).

Supplemental Note 5: Lattice distortions for the interfacial DMI.

Firstly, in order to study the lattice distortion, we quantitively analyzed the octahedral rotations/distortions near the perovskite and Ruddlesden-Popper interfaces (Supplementary Fig. 12). However, no distinguishable octahedral rotation exists at either interface. Therefore, we further calculated the layer-by-layer tetragonality (c/a ratio) based on HAADF images at room temperature, which provides higher signal-to-noise ratio and better contrast in the interface. As shown in Supplementary Fig. 13a and 13b, the variations of c/a ratio in I_3/T_6 superlattice illustrate different lattice distortions near the Ruddlesden-Popper and perovskite interfaces. An enhanced difference of lattice distortions between Ruddlesden-Popper and perovskite interfaces occurs in the $(Sr_2IrO_4)_3/(BaTiO_3)_6$ ($(SIO)_3/(BTO)_6$, I_3/B_6) superlattice (Supplementary Fig. 13d and 13e). BTO at the perovskite interface approximately maintains the tetragonality with c/a ratio of ~1.033, while the c/a ratio at the Ruddlesden-Popper interface is ~0.992 due to the geometric conditions at the interface of Ruddlesden-Popper structures[10].

In addition, we also try to measure the off-center displacements of TiO columns with respect to the geometric mass center of the surrounding four Sr/Ba columns (Supplementary Fig. 13f). The polarization of ultrathin BTO is much lower than the BTO bulk[11,12]. This will inevitably lead to a very small atomic displacement, which may be below the scanning noise and random specimen drift in acquiring STEM images (typically ~5 pm)[13]. Even so, we still observe a very little asymmetry of the off-center displacements in BTO/SIO superlattice as seen in Supplementary Fig. 13f, which is negligible in STO/SIO superlattice in Supplementary Fig. 13c.

The above structural analysis is summarized in Supplementary Fig. 14, the asymmetry of lattice distortions (c/a ratios) at both interfaces and the observation of very small polar distortions may provide a prerequisite of the interfacial DMI[5].

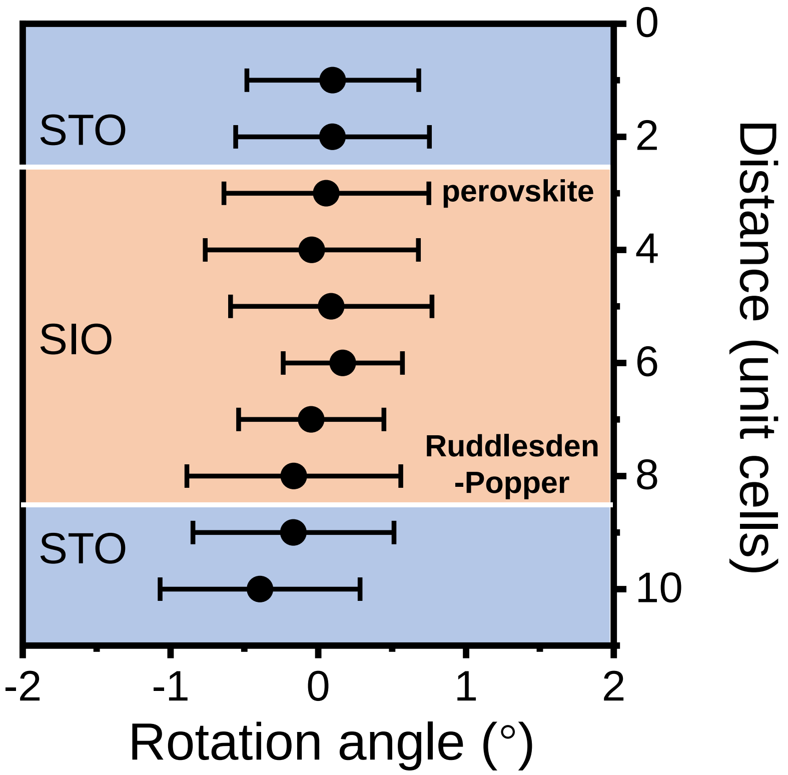

Supplementary Figure 12 | Negligible octahedral rotations/distortions in I_3/T_6 superlattice. The error bars are standard error of statistics.

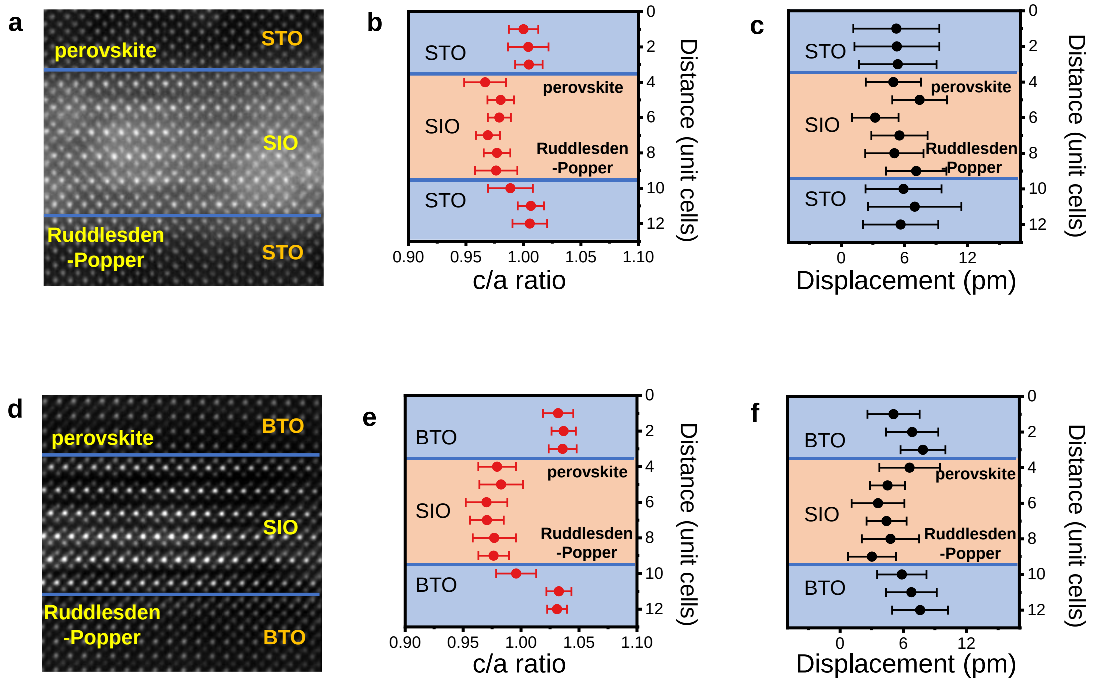

Supplementary Figure 13 | Quantitative analysis the layer-by-layer lattice distortion at room temperature. a,d, HAADF images of I_3/T_6 and I_3/B_6 superlattices. **b,e,** The magnitude mapping for tetragonality (c/a ratio) in I_3/T_6 and I_3/B_6 superlattices respectively. **c,f,** Off-center displacement in I_3/T_6 and I_3/B_6 superlattices. All the error bars are standard error of statistics.

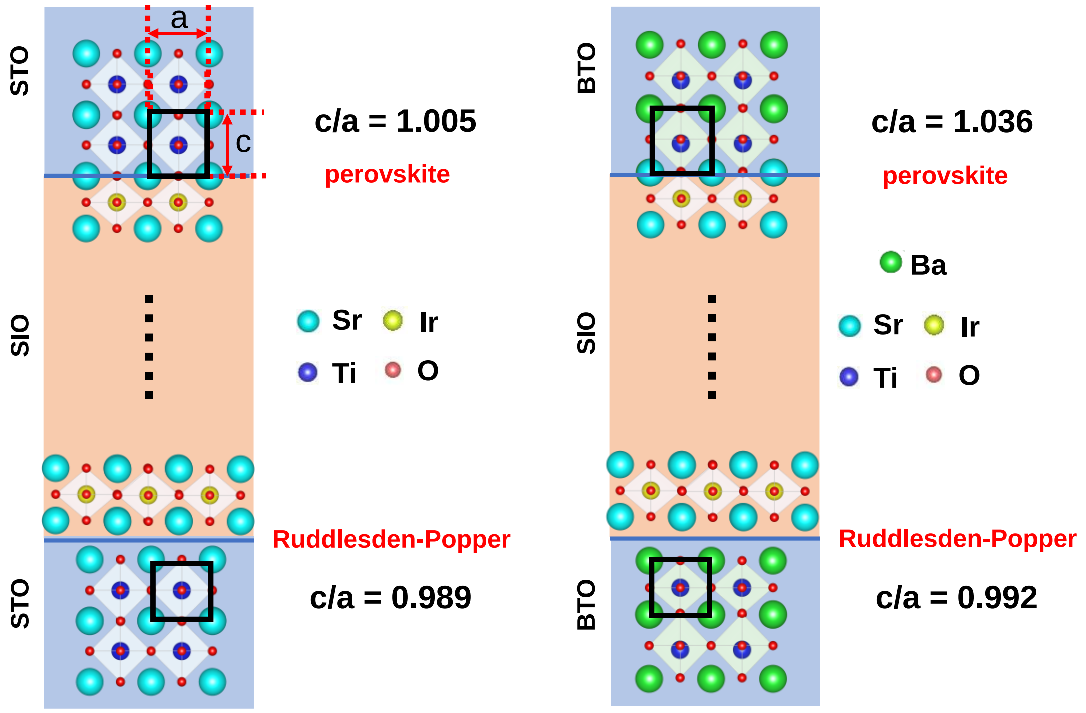

Supplementary Figure 14 | Schematic of the lattice distortion from the perovskite to the Ruddlesden-Popper interfaces.

References

1. Terashima, T. & Bando, Y. Reflection high-energy electron diffraction oscillations during epitaxial growth of high-temperature superconducting oxides. *Phys. Rev. Lett.* **65**, 21 (1990).
2. Manchon, A., Koo, H. C., Nitta, J., Frolov, S. M. & Duine, R. A. New perspectives for Rashba spin-orbit coupling. *Nat. Mater.* **14**, 9 (2015).
3. Zhang, W. et al. Electrical field enhanced interfacial Dzyaloshinskii-Moriya interaction in MgO/Fe/Pt system. *Appl. Phys. Lett.* **113**, 122406 (2018).
4. Kevin, S. et al. Temperature Dependent Brillouin Light Scattering Spectra of Magnons in YIG and Permalloy. *Phys. Rev. B* **96**, 024448 (2017).
5. Banerjee, S., Rowland, J., Erten, O. & Randeria, M. Enhanced Stability of Skyrmions in Two Dimensional Chiral Magnets with Rashba Spin-Orbit Coupling. *Phys. Rev. X* **4**, 031045 (2014).
6. Mermin, N. D. & Wagner, H. Absence of ferromagnetism or antiferromagnetism in one- or two dimensional isotropic Heisenberg models. *Phys. Rev. Lett.* **17**, 22 (1966).
7. Kosterlitz, J. M. & Thouless, D. J. Ordering, metastability and phase transitoins in two-dimensional systems. *J. Phys. C: Solid State Phys.*, **6**, 1181 (1973).
8. Shinobu, H. & Toshihiko, T. Phase Transition of Quasi-Two Dimensional Planae System. *Progress. Theor. Phys*. **63**, 387-401 (1980).
9. Di, K. et al. Direct Observation of the Dzyaloshinskii-Moriya Interaction in a Pt/Co/Ni Film. *Phys. Rev. Lett.* **114**, 047201 (2015).
10. Tian, W. & Pan X. Q. Transmission electron microscopy study of n = 1-5 $Sr_{n+1}Ti_nO_{3n+1}$ epitaxial thin films. *J. Mater. Res.* **17**, 2013-2026 (2001).
11. Junquera, J. & Ghosez, P. Critical thickness for ferroelectricity in perovskite ultrathin films. *Nature* **422**, 506-509 (2003).
12. Wen, Z., Li, C., Wu, D., Li, A. & Ming, N. Ferroelectric-field-effect-enhanced electroresistance in metal/ferroelectric/semiconductor tunnel junctions. *Nat. Mater.* **12**, 617-621 (2013).
13. Sun, Y. et al. Subunit cell-level measurement of polarization in an individual polar

vortex. *Sci. Adv.* **5**, eaav4355 (2019).
